# Increased Frequency of CTLA-4 and PD-1 Expressing Regulatory T Cells and Basophils With an Activating Profile in Infants With Moderate-to-Severe Atopic Dermatitis Hypersensitized to Food Allergens

**DOI:** 10.3389/fped.2021.734645

**Published:** 2021-11-29

**Authors:** Agurtzane Bilbao, Raquel Pérez-Garay, Idoia Rius, Alex Irurzun, Iñigo Terrén, Ane Orrantia, Gabirel Astarloa-Pando, Francisco Borrego, Olatz Zenarruzabeitia

**Affiliations:** ^1^Immunopathology Group, Biocruces Bizkaia Health Research Institute, Barakaldo, Spain; ^2^Pediatrics Service, Cruces University Hospital, Barakaldo, Spain; ^3^Clinical Analysis Service, Cruces University Hospital, Barakaldo, Spain; ^4^Ikerbasque, Basque Foundation for Science, Bilbao, Spain

**Keywords:** atopic dermatitis, food sensitization, specific IgE, regulatory T cells, basophils, CD300 receptors, inhibitory receptors

## Abstract

**Background:** Infants with severe atopic dermatitis (AD) may be sensitized to foods that have not been introduced into their diet, posing a risk for developing an immediate hypersensitivity reaction on the first exposure to the food to which they are sensitized. The aim of this work was to perform an analysis of the sensitization profile in infants with moderate-to-severe AD and to identify cellular and molecular markers for food allergy (FA).

**Methods:** Blood samples from healthy donors and children with moderate-to-severe AD were studied. Specific IgE to several allergens were determined using ImmunoCAP FEIA system and ISAC technology. Furthermore, using flow cytometry-based studies, basophils and regulatory T (Treg) cells were phenotypically characterized.

**Results:** 90% of children with AD were sensitized to food antigens before introducing them into the diet, and 100% developed FA. Phenotypic analysis showed a significantly higher percentage of CTLA-4 and PD-1 expressing Treg cells in AD patients than in healthy controls. Basophils from patients exhibited a marked reduction in the expression of CD300a, higher expression of FcεRI and CXCR4, and to some extent higher expression of CD63 and CD300c.

**Conclusions:** Infants with moderate-to-severe AD are at high risk of being sensitized to food allergens. Therefore, to avoid allergic reactions, broad-spectrum sensitization studies are necessary before introducing complementary diet. Increased expression of CTLA-4 and PD-1 suggests greater suppressive potential of Treg cells in infants with AD than healthy controls. Furthermore, our results suggest a role for CD300 molecules on circulating basophils as possible biomarkers for FA susceptibility.

## Introduction

Atopic dermatitis (AD) is a common inflammatory skin disease of increasing prevalence that affects 7–14% of adults and 10–20% of children globally (1). The onset of AD occurs during the first year of life in ~60% of individuals and only the 10% of affected children start the disease after the age of 7 (2). It causes generalized skin dryness and eczematous lesions constituted by spongiosis, edema, and microvesicles, which give rise to itching, skin irritation, scratching, and symmetrically distributed inflammatory lesions (2, 3). Although the severity of the disease is variable, it is moderate-to-severe in up to one third of patients, many of whom require systemic treatment.

Several epidemiological studies have established that AD is often accompanied by allergic rhinitis, asthma and food allergy (FA) (the “atopic march”), as well as conjunctivitis (4–7). Additionally, although the mechanisms are still unknown, it has been shown that infants with severe AD may be sensitized to foods that have not yet been introduced into their diet (8–11), turning these children into high-risk patients when introducing new foods. In this sense, Palmer et al. observed in 2013 that a high proportion (36%) of children with moderate-to-severe AD were already sensitized to egg at 4 months of age before introducing solid foods in their diet (8). Moreover, there are studies showing that the early introduction of solid food at 4–6 months of age reduces the risk of developing FA in patients with severe AD not yet sensitized (12–14), which is changing the pattern of complementary feeding in children with AD. Despite these studies, in the everyday practice of hospitals in our geographic area only milk and egg specific immunoglobulin E (sIgE) are determined in infants with AD. As sensitization can affect other foods in addition to eggs and milk, we considered very relevant to extend the determination of sIgEs to other foods before starting complementary feeding.

Allergy is the result of a dysregulation of immune tolerance, that is, the immune system recognizes as dangerous a substance that is harmless to the body. One of the most important cells in the regulation of the immune system are regulatory T (Treg) cells, which play a major role in the regulation of allergic reactions by inducing and maintaining immune tolerance to allergens (15–18). Treg cells play an important role in various immune pathologies (15, 17, 19–22). Thus, it has been observed that the frequency of Treg cells is altered both in allergic patients and in patients with AD, compared to healthy individuals (23–28). Although others have found no significant differences in the frequency of blood Treg cells between patients and healthy controls (29–33).

The immune response is also regulated, among others, by activating and inhibitory receptors expressed on the surface of immune cells. In this sense, the involvement of CD300 surface receptors in the regulation of allergic responses has been demonstrated (34–39). It is known that CD300a expressed on basophils suppresses the basophil anaphylactic degranulation by its interaction with phosphatidylserine and phosphatidylethanolamine exposed on apoptotic cells (34, 35), whereas CD300c works as a costimulatory molecule during immunoglobulin E (IgE)-mediated basophils activation (37). Moreover, in basophils from allergic individuals, a lower expression of CD300a inhibitory receptor (34) and a higher expression of CD300c activating receptor (37, 38) have been described. In AD patients a significant increase in CD300a total expression was observed in biopsies from lesional skin compared to normal skin, specifically on eosinophils, macrophages, and mast cells (40). However, CD300 expression in basophils from AD patients has not been studied to date.

In this pilot study, we have done a comprehensive analysis of the sensitization profile of infants with moderate-to-severe AD before the introduction of complementary feeding. Moreover, we have performed immune profiling analysis and conducted surveys about the eating habits of families with the aim to understand possible mechanisms by which these children become sensitized at such an early age, and finally we have determined the development of FA by the age of two.

## Methods

### Donors and Samples

Blood samples from healthy donors and infants with moderate-to-severe AD were collected prospectively through the Basque Biobank (http://www.biobancovasco.org), which complies with the quality management, traceability, and biosecurity set out in the Spanish Law 14/2007 of Biomedical Research and the Royal Decree 1716/2011. Parents of all subjects provided written and signed informed consent in accordance with the Declaration of Helsinki. The protocol was approved by the Ethics Committee for Clinical Research of Cruces University Hospital (CEIC E18/26).

As healthy controls (*n* = 9), children under 2 years of age who had not presented episodes of AD during their lifetime, did not have a diagnosis of allergy, and had not presented allergic symptoms to potentially allergenic foods were recruited. AD patients (*n* = 10) were under 12 months of age when they were included in the study and they were not being treated with corticosteroids at the time of blood sampling. The inclusion criterion was a diagnosis of AD in the first 6 months of life, either for lesions on the face and/or large areas that had required topical corticosteroids for more than 7 days a month (moderate AD) or generalized lesions that had been treated with a course of systemic corticosteroids (severe AD) (more details about inclusion criteria and clinical features are detailed in Supplementary Material). Potentially allergenic foods such as milk, egg, hake, cod, tuna, salmon, shrimp, mackerel, rooster, peanut, hazelnut, almond, nut, pistachio, cashew, wheat, soy, rice, potato, lentil, white bean, chickpea, kiwi, or peach had not been introduced into their diet when they were recruited for the study.

Food sensitization was diagnosed by *in vitro* sIgEs quantification that was made using the ImmunoCAP FEIA system, as specified below. sIgE values > 0.35 KU/L were considered positive. Regarding FA development, the evolution of the children was followed for 2 years approximately (Supplementary Table 1). During the monitoring, *in vitro* sIgEs quantifications and/or skin prick test in response to specific allergens were carried out. Skin tests were regarded as positive if the wheal diameter was >3 mm, and food has been introduced in case of prick test < 3 mm. A positive control of histamine 10 mg/mL and a negative control of saline 0.9% were included. At 2 years of age, children with an exclusion diet were considered allergic to food. Food provocation was not justified, given the high predictive value of their degree of sensitization assessed by prick test and serum sIgE values (41–44) (more details in Supplementary Material).

### Specific IgEs Quantification

sIgEs to cow's milk, egg white and yolk, hake, cod, tuna, salmon, shrimp, mackerel, rooster, peanut, hazelnut, almond, nut, pistachio, cashew, wheat, soy, rice, potato, lentil, white bean, chickpea, kiwi, peach and to specific allergens alfalactalbumin (ALA), betalactoglobulin (BLG), casein, ovalbumin (OA), ovomucoid (OM), Ara h1, Ara h2, Ara h3, Ara h 8, Ara h 9, Cor a1, Cor a8, rJug r1, rJug r3, rPru p1, rPru p4, rPru p3, rAct d8, rGad c1, nGly m4, nGly m5, and nGly m6 were determined with the ImmunoCAP FEIA system (Thermo Fisher Scientific) according to the manufacturer's instructions. Values higher than 0.35 kU/L were considered positive.

### Immuno Solid-Phase Allergen Chip

IgE sensitization was also analyzed with a customized allergen chip based on the Immuno Solid-phase Allergen Chip (ISAC) technology (Thermo Fisher Scientific). Healthy donors and patients were tested for a panel of 112 allergen components (Supplementary Table 2), according to the manufacturer's instructions. Briefly, allergen components (triplicates) were immobilized on a glass slide. The assay includes a two-step reaction: (1) 30 μl of serum sample were applied onto the microarray reaction site; and (2) after incubation and washing, fluorescence-labeled anti-human IgE detection antibody was applied. After incubation, washing and drying, the microarrays were analyzed using a confocal laser scanner (LuxScan 10K Microarray Scanner) and evaluated by Phadia MIA Microarray Image Analyzer version 1.4.3 software (Thermo Fisher Scientific). For calibration and detection of background signals, a calibrator serum and sample diluent from Thermo Fisher Scientific were included in each analysis. The results were expressed as ISAC standardized units (ISU), and values ≥ 0.1 ISU were considered positive.

### Flow Cytometry Analyses

Whole blood samples from healthy donors and AD patients were collected in sodium citrate containing tubes and were used for phenotypical characterization of basophils and Treg cells. For the characterization of basophils, 100 μl of whole blood was stained with fluorochrome conjugated mAbs for 20 min at room temperature (RT). Next, red blood cells were lysed using 1X BD FACS Lysing buffer for 15 min at RT. Then, cells were washed with phosphate buffered saline (PBS) to remove unbound mAbs and further acquired in a MACSQuant^®^ Analyzer 10 Flow Cytometer (Miltenyi Biotec). As a negative control, we used fluorescence minus one (FMO) and unstained controls. FMO control contains all the fluorochrome conjugated mAbs with the exception of the marker of interest and it is used to identify and gate cells in the context of data spread due to the multiple fluorochromes in a given panel.

For the characterization of Treg cells, first peripheral blood mononuclear cells (PBMCs) were freshly isolated from donors' whole blood by Ficoll Paque Plus (GE Healthcare) density gradient centrifugation. PBMCs were incubated with LIVE/DEAD Fixable Near-IR Dead Cell Stain reagent (Invitrogen) before the extracellular staining in order to detect dead cells following the manufacturer's protocol. For extracellular staining, PBMCs were incubated with the specific mAbs for 30 min at 4°C in the dark. Cells were washed with PBS containing 2.5% of bovine serum albumin (BSA) (Sigma-Aldrich) and were permeabilized and fixed using Foxp3/Transcription Factor Staining Buffer Set (eBioscience) following manufacturer's instructions. Then, they were incubated with APC anti-Foxp3 and PerCP-Cy5.5 anti-Helios during 30 min at RT in the dark. Lastly, cells were washed, resuspended in 200 μl of PBS and acquired in a MACSQuant^®^ Analyzer 10 Flow Cytometer. Flow cytometry data were analyzed using FlowJo [version 10.0.7 (TreeStar)] and FlowLogic (version 7.3) software. The flow cytometry panels used in this study are shown in Supplementary Table 3.

### Statistical Analysis and Graphical Representation

GraphPad Prism software (version 9) was used for graphical representation and statistical analysis. Data were represented in scatter plots with bars showing the means ± standard error of the mean (SEM). For comparison between healthy and DA patients, the non-parametric, unpaired Mann–Whitney rank test was used. ^*^p < 0.05, ^**^p < 0.01, ^***^p < 0.001, and ^****^p < 0.0001.

The detailed list of antibodies, allergens of the Immuno Solid-phase Allergen Chip (ISAC) and flow cytometry analyses are detailed in the Supplementary Material section.

## Results

### Children With Moderate-to-Severe AD Are Very Likely to Be Hypersensitized to Food Antigens

With the aim of knowing the sensitization profile of infants with moderate-to severe AD before starting with complementary diet, we determined sIgEs to several food allergens using ImmunoCAP FEIA system. In addition, determination of sIgE for 112 allergens from 50 allergenic sources (43 foods) was also performed by ISAC. Nine out of 10 (90%) children with moderate-to-severe AD showed sIgE against some of the tested antigens (Table 1), while around 33% of healthy control children were sensitized to food antigens, although to a significantly lower number of allergens (Table 2).

**Table 1:** sIgEs determined by ImmunoCAP FEIA and ISAC in infants with moderate-to-severe AD.

**Patient**	**ImmunoCap (>0.35 KU/L)**	**ISAC >0.3 ISU-E**	**Age**
DA001	Negative	Negative	3 months
DA003	Egg white (2.6); ovomucoid (0.37); ovalbumin (2.19); peanut (1.07); rArah1 (0.86)	Negative	11 months
DA004	Egg white (0.53); ovalbumin (0.46)	nd^*^	4 months
DA005	Egg white (5.78); ovomucoid (7.19); ovalbumin (0.69); peanut (6.05); rAra h2 (5.95); almond (0.72); hake (8.8)	Ovomucoid (1.3); rAra h2 (1.8)	11 months
DA006	Egg (55.50); egg white (66.40); ovomucoid (>100); ovalbumin (23.50); cow milk (8.42); α-lactoalbumin (0.47); β-lactoglobulin (1.25); casein (9.64); peanut (25.20); rAra h1 (10.90); rAra h2 (1.42); rAra h3 (2.64); hazelnut (16.60); nCor a9 (15.80); almond (8.38); potato (50.20); wheat (6.77)	Ovomucoid (12); ovalbumin (6.9); casein (1.5); rAra h1 (3.2); rAra h2 (7.2)	6 months
DA007	Egg (11.70); egg white (10.20); ovomucoid (9.73); ovalbumin (4.69); cow milk (3.84); casein (0.83); peanut (0.87); rAra h1 (1.46); rAra h9 (0.66); almond (1.68); oat (2.71); wheat (6.50)	Ovomucoid (0.7)	3 months
DA008	Cow milk (2.70); casein (1.65); ovalbumin (1.7); ovomucoid (27.70); potato (3.52); kiwi (8.09)	Ovomucoid (8.5); α-lactoalbumin (1.4); casein (1.3)	6 months
DA009	Ovalbumin (1.25); hake (0.71); rGad c1 Cod (0.96)	Negative	9 months
DA010	Ovalbumin (6.22); almond (0.43); wheat (1.02)	Ovalbumin (2)	2 months
DA011	Cow milk (1.97); casein (1.32); ovalbumin (1.90); potato (0.54)	Ovalbumin (0.4); β-lactoglobulin (1.3); casein (1)	4 months

* *nd, no data available*.

**Table 2:** sIgEs determined by ImmunoCAP FEIA and ISAC in healthy control children.

**Patient**	**ImmunoCap (>0.35 KU/L)**	**ISAC > 0.3 ISU-E**	**Age**
CDA001	Negative	Negative	1 year
CDA002	Egg white (0.66); ovalbumin (0.9)	Egg white (1.5)	7 months
CDA003	Negative	Negative	2 years
CDA006	Cow milk(1.38); ovalbumin (0.66); ovomucoid (0.61); casein (0.76)	Ovomucoid (1.1); α-lactoalbumin (0.5); casein (0.5)	9 months
CDA007	Negative	Negative	1 year
CDA008	Negative	Negative	1 year
CDA010	Negative	Negative	2 months
CDA012	Negative	Negative	3 months
CDA013	Egg white (1.21)	Negative	7 months

Sensitization does not always equate to clinical allergy. In our study cohort, based on their clinical symptoms history, serum sIgE levels, and/or skin prick test results, while none of the healthy control children were diagnosed of FA, all 10 children that had been diagnosed with moderate-to-severe AD were found to be allergic to some food by the age of 2, and 70% of them were allergic to two or more foods. Although, the vast majority were allergic to eggs and/or milk, it is very important to point out that five of them (50%) were allergic to nuts, two (20%) were allergic to fish and one was allergic to kiwi (Supplementary Table 1). These results indicate that, in moderate-to-severe AD children, the determination of only sIgE to milk and egg before starting with the complementary diet is not enough to prevent allergic reactions that can become very serious.

### Families Eating Habits Are Associated With the Sensitization Profile of Children With Moderate-to-Severe AD

In order to elucidate the possible mechanisms by which children with moderate-to-severe AD could be sensitized, we first analyzed whether the mothers had the same sIgEs as their children that could suggest a transmission either during pregnancy and/or through breastfeeding (45, 46). We could only obtain samples from four mothers, but only one had dust mite sIgEs, while her child did not (data not shown). We also studied the eating habits of people living with AD children to determine whether the sensitization profile correlated with food allergens that may be present in the home environment. Surveys of families revealed that sensitization to specific antigens in children was associated to foods commonly consumed not only by the mother but also by other members of the family unit.

### Cellular and Molecular Markers in AD Children

#### Regulatory T Cells From Moderate-to-Severe AD Children

**Figure 1:**
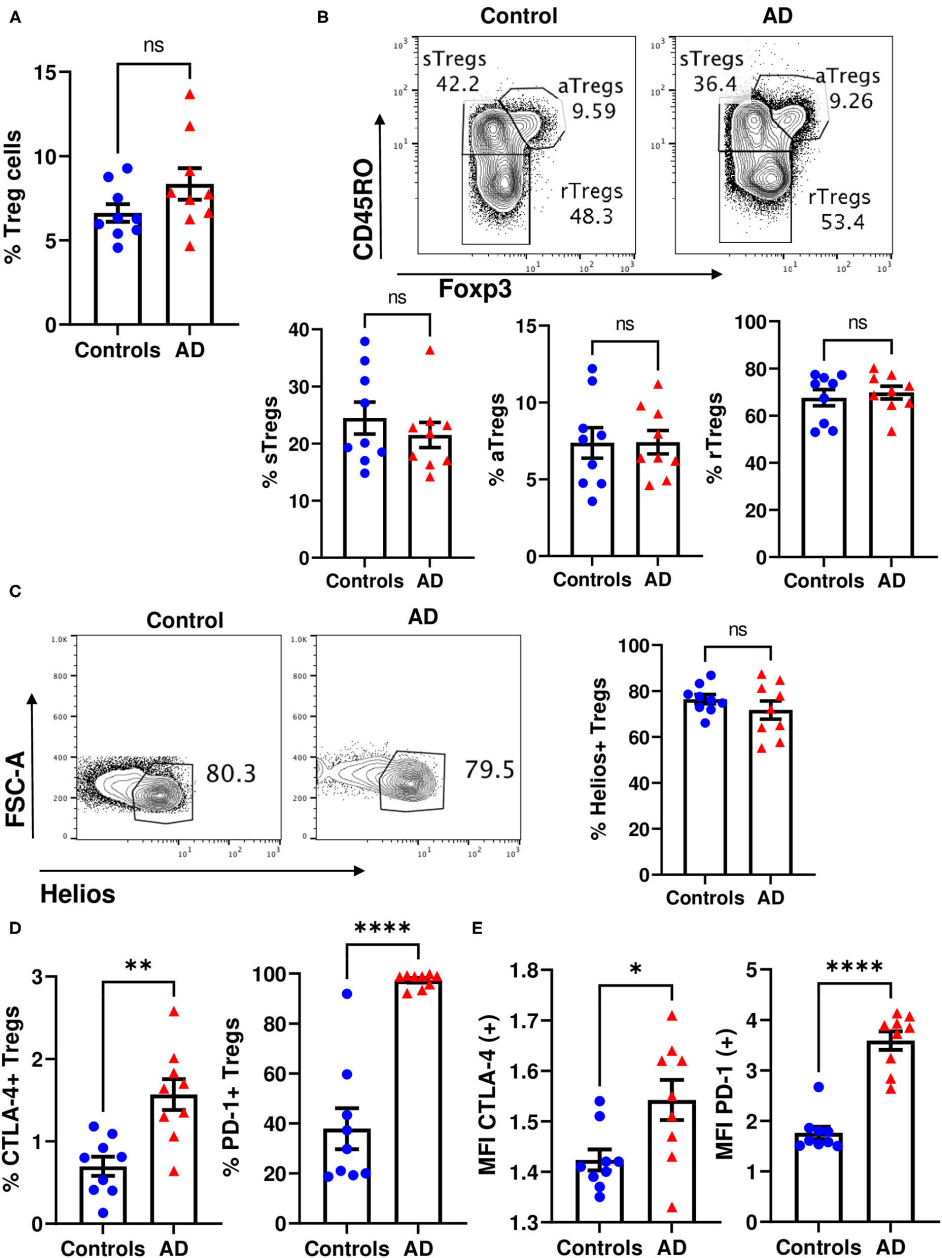
Phenotypic characterization of Treg cells from moderate-to-severe AD children. **(A)** Dot-bar graphs showing the frequency of total Treg cells identified as CD4+CD25highFoxP3+ cells in healthy control (blue dots) and moderate-to-severe AD (red triangles) children. **(B)** Representative contour plots showing the percentages of activated Treg cells (aTregs) (CD45RO^+^Foxp3^high^), resting Treg cells (rTregs) (CD45RO^−^Foxp3^low^), and cytokine-secreting Treg cells (sTregs) (CD45RO^+^Foxp3^low^) in healthy control (left) and moderate-to-severe AD (right) children (upper line) and dot-bar graphs indicating the frequency of different Treg cells subsets (lower line). **(C)** Representative contour plots showing the percentages of Helios+ Treg cells in healthy control and moderate-to-severe AD children and dot-bar graph indicating the frequency of Helios+ Treg cells in healthy control (blue dots) and moderate-to-severe AD (red triangles) children. **(D)** Dot-bar graphs representing the percentage of CTLA-4 expressing Treg cells and of PD-1 expressing Treg cells in healthy control (blue dots) and moderate-to-severe AD (red triangles) children. **(E)** Dot-bar graphs showing the MFI of CTLA-4 and PD-1 in positive cells for each receptor. Each dot represents a donor, means ± SEMs are shown. Mann–Whitney test. ns, no significant, *p < 0.05, **p < 0.01, and ****p < 0.0001.

Peripheral blood circulating Treg cells can be identified as CD4+CD25highFoxP3+ cells or as CD4+CD25highCD127- cells (Supplementary Figures 1A,B). Compared to healthy controls, the analysis did not reveal significant differences in the frequency of total Treg cells in patients with moderate-to-severe AD (Figure 1A and data not shown). To further explore the differences, we performed analyses of Treg cell subsets. Based on the expression of Foxp3 and CD45RO, Treg cells were classified into activated Treg cells (aTregs) (CD45RO+Foxp3high), resting Treg cells (rTregs) (CD45RO-Foxp3low) and cytokine-secreting Treg cells (sTregs) (CD45RO+Foxp3low) (Figure 1B). Moreover, in order to differentiate peripherally derived Treg cells (Helios-) from thymic derived Treg cells (Helios+) the expression of the transcription factor Helios was analyzed (Figure 1C). We did not observe significant differences in the frequency of each subpopulation between moderate-to-severe AD and healthy control groups (Figures 1B,C).

Human Treg cells are characterized by the expression of inhibitory molecules such as programed cell death-1 (PD-1) and cytotoxic T-lymphocyte antigen-4 (CTLA-4) (21, 22, 47–50). We observed a statistically significant increase in the percentage of CTLA-4 and PD-1 expressing cells in moderate-to-severe AD patients compared to healthy controls (Figure 1D). Moreover, in CTLA-4 positive cells and in PD-1 positive cells the median fluorescence intensity (MFI) of the expression of each receptor was significantly higher in AD patients than in healthy controls (Figure 1E).

#### Phenotypic Characterization of Basophils From Moderate-to-Severe AD Children

**Figure 2:**
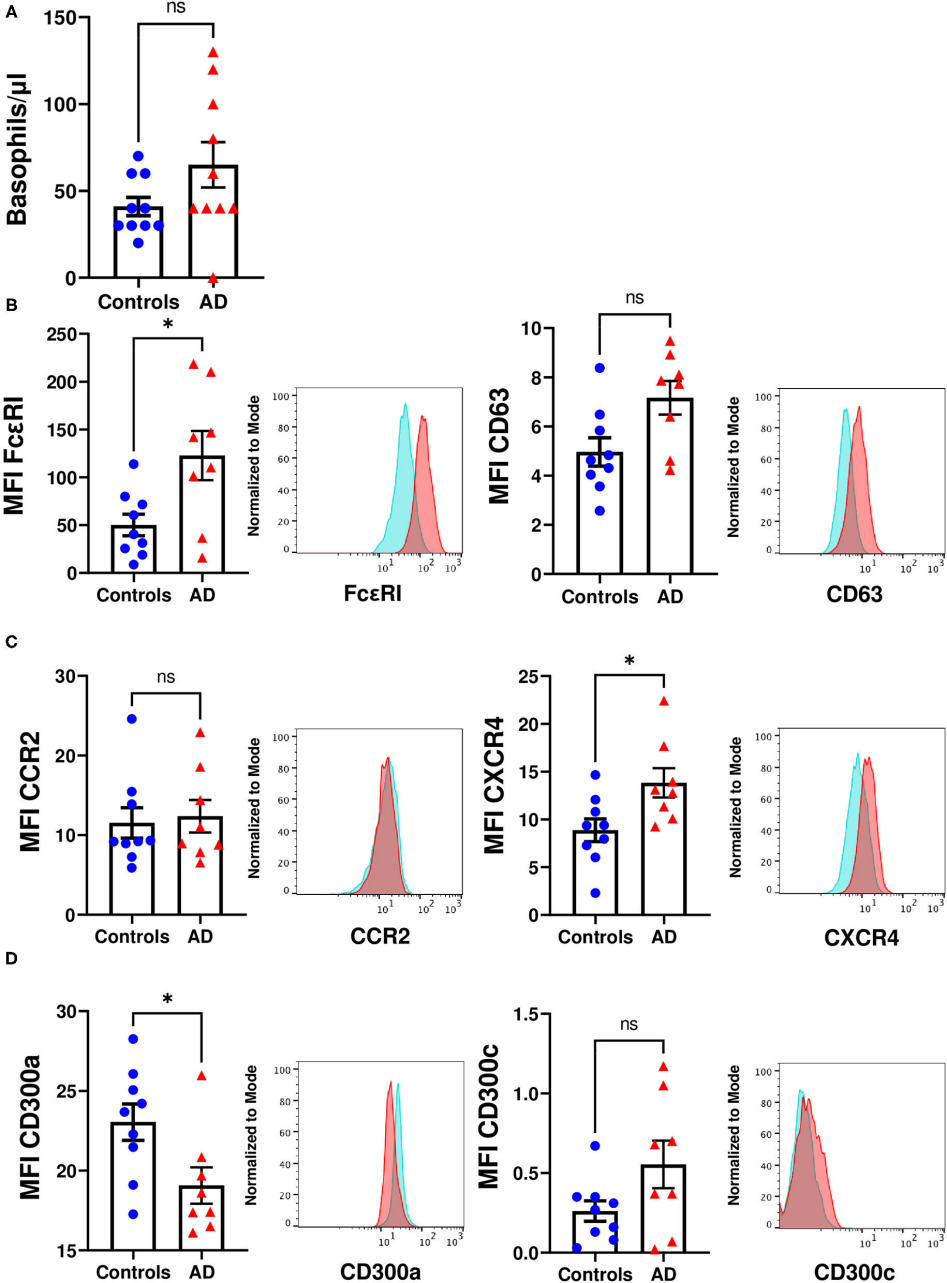
Phenotypic characterization of basophils from moderate-to-severe AD children. **(A)** Dot-bar graphs showing the number of basophils/ul in healthy control (blue dots) and moderate-to-severe AD (red triangles) children's whole blood. **(B–D)** Dot-bar graphs showing MFI of FcεRI, CD63, CCR2, CXCR4, CD300a, and CD300c on circulating basophils from healthy control (blue dots) and moderate-to-severe AD (red triangles) children and histograms of representative individuals (healthy control in blue and AD patients in red). Each dot represents a donor, means ± SEMs are shown. Mann–Whitney test. ns, no significant, *p < 0.05.

Next, we performed an analysis of blood circulating basophils. We did not observe significant differences in the number of blood basophils between patients with moderate-to-severe AD and healthy controls (Figure 2A). Our results showed that, compared to healthy individuals, basophils from moderate-to-severe AD children expressed significantly higher expression levels of FcεRI, and that the expression of the basophil activation marker CD63 also tended to be higher (Figure 2B), in accordance with previously reported results in basophils from allergic individuals (38). Human basophils express, among others, the chemokine receptors CCR2, which positively regulates basophil degranulation in response to monocyte chemoattractant protein-1 (MCP-1), and CXCR4, important for basophil activation and recruitment to inflammatory sites (51, 52). We observed that the expression of CXCR4 was significantly increased in AD patients compared to healthy children (Figure 2C). Lastly, we analyzed the expression of CD300 molecules, surface receptors known to regulate the activation threshold of human basophils in response to IgE (39). As it has been previously described in allergic individuals (34, 37, 38), the surface expression of CD300a was significantly lower in moderate-to-severe AD patients than in control children while the expression of CD300c tended to be higher (Figure 2D). Taking together, although our analysis did not reveal significant differences in the number of blood basophils, patients with moderate-to-severe AD exhibited basophils with a more activating profile, reminiscent of basophils from allergic individuals.

## Discussion

In this study we have observed that children with moderate-to-severe AD had sIgEs against multiple food antigens before introducing them into their diet, which emphasizes the need for caution when first initiating complementary diet to these children.

It has been recently described that murine fetal mast cells mediate postnatal allergic responses dependent on maternal IgE (53). Moreover, it has been also demonstrated that allergen sIgEs are transmitted from maternal blood to breast milk, which suggests that the transmission of sIgEs from mothers to offspring *via* milk may affect the development of allergy in infants (45). Although we compared the sensitization profile of the children with that of the mothers, we did not find similarities in any of the studied cases, indicating that, in our cohort, sensitization was not dependent on maternal IgE. Nevertheless, more studies are required to rule out this possibility.

In recent years, increasing evidence regarding the importance of allergic sensitization through the skin are accumulating (4, 9, 12, 54–56). In this sense, we have observed that sensitization to specific antigens in children with moderate-to-severe AD was associated to foods commonly consumed not only by the mother, but also by other members of the family unit, suggesting that the skin could be one of the routes of sensitization. It has been well-established in animal studies that sensitization to food allergens can occur through the skin, giving rise to IgE-mediated hypersensitivity responses (4, 57–60). Not only in animal models, there is also clinical data supporting the dual allergen exposure hypothesis. The Avon Longitudinal Study of Parents and Children (ALSPAC) birth cohort study demonstrated that infants with peanut allergy by the age of 5 years were more likely to have had severe AD in the first 6 months of life and to have been treated with peanut oil for dry skin (9). Based on evidences from mouse models and clinical studies, it has been suggested that, if the skin barrier can be improved and/or the inflammation of AD can be proactively prevented, in combination with early introduction of food antigens, the incidence of FA and possibly other forms of allergic diseases might be decreased (55, 56, 60–62).

Regulatory mechanisms are necessary for the immune system to maintain peripheral tolerance and Treg cells are the most important cells involved in the immune system regulation. Tregs achieve their immunosuppressive function both through direct cell-to-cell contact (by CTLA-4, LAG-3, PD-1, and FasL) and through the secretion of immunoregulatory cytokines such as IL-10 and TGF-β. Some of the known functions of Treg cells are to inhibit the activation of Th2 cells by suppressing the production of IL-2, IL-5, IL-9, and IL-13, to block the migration of effector T cells into inflamed tissues, to suppress the production of IgE, to induce the production of IgG4 and to limit Th17-mediated inflammation (16, 21, 28, 63, 64). Whether Treg cells frequencies are altered in AD patients has been addressed by several studies. While some revealed an increase in the frequency of circulating Treg cells in AD patients compared to healthy controls, in others similar expansion has been reported (25, 26, 28, 31, 33). Also in allergy, some authors described a reduction on the frequency of Treg cells in blood (23, 24, 27) and others state that the frequency is similar in allergic and non-allergic individuals (29, 30, 32). We did not observe significant differences between moderate-to-severe AD infants and healthy controls in the percentage of Treg cells and neither in the frequency of the different subpopulations. However, the phenotypic analysis of Treg cells showed a significantly higher expression of the inhibitory molecules CTLA-4 and PD-1 in AD patients. A higher expression of CTLA-4 has been previously demonstrated in Treg cells from severe AD patients (28). PD-1 and CTLA-4 are two Treg cells functional markers involved in cell-to-cell communication between Treg and target cells. It has been suggested that CTLA-4 expression levels may indicate the suppressive potency of Treg cells (47, 48), while the expression of PD-1 has been associated not only with the suppressive capacity (21, 49, 65) but it has also been considered an activation marker of Treg cells (22, 50). Therefore, the increased expression of CTLA-4 and PD-1 in Treg cells from moderate-to-severe AD infants suggests that they are more activated and have a higher suppressive potency.

Multiple studies in the last few years have highlighted the importance of CD300 molecules in several pathological conditions (39, 66–68). Hence, it has been described previously that the expression of the inhibitory receptor CD300a is modulated in AD patients and that it could influence the inflammatory response (40). In fact, a significant increase in CD300a total expression was observed in AD biopsies from lesional skin when compared to normal skin (40). In the same line, several studies in allergic patients have suggested a key role of CD300 molecules in the modulation of the activation threshold of basophils, eosinophils, and mast cells, during allergic reactions (39). In fact, based on previously published evidences, we have recently suggested a model describing how CD300 activating and inhibitory receptors regulate IgE-dependent basophils activation (37, 39). Here, we have observed that basophils from patients with moderate-to-severe AD infants exhibited a marked reduction in the expression of CD300a and a slightly higher expression of CD300c than healthy controls, suggesting that these basophils have a more activating profile, reminiscent of basophils from allergic individuals. In fact, when we analyzed allergy incidence at 2 years of age we observed that all children included in the study had developed food allergy, being 70% allergic to two or more foods, suggesting that the expression levels of CD300a and CD300c on basophils may indicate an allergic predisposition in moderate-to-severe AD infants.

Finally, the CXCR4/CXCL12 signaling axis plays a pivotal role in numerous biological processes and an overexpression of CXCR4 has been associated with several diseases including skin inflammatory diseases such as psoriasis and AD (69, 70). In this sense, it has been recently demonstrated that an endogenous CXCR4 antagonist reduced skin inflammation in an AD mouse model, demonstrating a role for the CXCR4/CXCL12 axis in AD, as well as showing a potential role for CXCR4-antagonizing agents as therapeutic options in skin inflammatory diseases (70). In addition, higher serum levels of CXCL12 were found in AD patients compared aged-matched healthy controls (71). Here, we have described for the first time an increased expression of CXCR4 on basophils from infants with moderate-to-severe AD compared to healthy controls, which could suggest a greater recruitment potential of basophils to the site of inflammation. However, further studies are needed in order to understand the functional significance and clinical relevance of the increased expression of CXCR4 on circulating basophils from infants with moderate-to-severe AD and its relationship with allergic susceptibility.

Altogether, our results show that infants with moderate-to-severe AD are at very high risk of being sensitized to food allergens and highlight the need for broad-spectrum food sensitization studies in order to personalize the introduction of complementary foods and thus avoid allergic reactions that could become serious. We have observed an increment in the expression of CTLA-4 and PD-1 in Treg cells from AD patients, suggesting a higher suppressive activity. Moreover, our results suggest a role for CD300 molecules on circulating basophils as biomarkers for FA susceptibility.

## Data Availability Statement

The raw data supporting the conclusions of this article will be made available by the authors, upon reasonable requests.

## Ethics Statement

The studies involving human participants were reviewed and approved by Ethics Committee for Clinical Research of Cruces University Hospital (CEIC E18/26). Written informed consent to participate in this study was provided by the participants' legal guardian/next of kin.

## Author Contributions

OZ and AB conceived the project. OZ and FB designed experiments. AB, RP-G, and IR obtained the clinical samples and clinical data from AD patients. AB and RP-G obtained samples and clinical data from healthy controls. AI interviewed families on eating habits. OZ performed experiments, data analysis, compiled figures, and wrote the manuscript. FB, IT, AO, and GA-P provided intellectual input. All authors critically reviewed the manuscript.

## Funding

This study has been funded by the Department of Health, Basque Government through the Project 2019111031 and by a grant from the Agencia Estatal de Investigación Project PID2019-109583RB-I00/AEI/10.13039/501100011033. OZ is recipient of a postdoctoral contract funded by Instituto de Salud Carlos III-Contratos Sara Borrell 2017 (CD17/0128) and the European Social Fund (ESF)-The ESF invests in your future. IT is recipient of a predoctoral contract funded by the Department of Education, Basque Government (PRE_2020_2_0007). AO is recipient of a fellowship from the Jesús de Gangoiti Barrera Foundation (FJGB20/007). GA-P is the recipient of a fellowship from the BBK Fundazioa (BBK/PRACT/20/001). FB is an Ikerbasque Research Professor, Ikerbasque, Basque Foundation for Science.

## Conflict of Interest

The authors declare that the research was conducted in the absence of any commercial or financial relationships that could be construed as a potential conflict of interest.

## Publisher's Note

All claims expressed in this article are solely those of the authors and do not necessarily represent those of their affiliated organizations, or those of the publisher, the editors and the reviewers. Any product that may be evaluated in this article, or claim that may be made by its manufacturer, is not guaranteed or endorsed by the publisher.
