# Supplementary material for: Increased Frequency of CTLA-4 and PD-1 Expressing Regulatory T Cells and Basophils With an Activating Profile in Infants With Moderate-to-Severe Atopic Dermatitis Hypersensitized to Food Allergens
Source: Front Pediatr. 2021 Nov 29;9:734645. doi: 10.3389/fped.2021.734645 (PMC8667617; doi:10.3389/fped.2021.734645)
Supplement: Supplementary file 1 [file Data_Sheet_1.pdf]

Supplementary Material for

Increased frequency of CTLA-4 and PD-1 expressing regulatory T cells and basophils with an activating profile in infants with moderate-to-severe Atopic Dermatitis hypersensitized to food allergens

Agurtzane Bilbao, Raquel Pérez-Garay, Idoia Rius, Alex Irurzun, Iñigo Terrén, Ane Orrantia, Gabirel Astarloa-Pando, Francisco Borrego, Olatz Zenarruzabeitia*

*Corresponding author. Email: olatz.zenarruzabeitiabelaustegui@osakidetza.eus

This file includes:

Methods section: Diagnosis criteria, patients´ clinical features and list of antibodies used for flow cytometric analysis.

Supplementary Table 1. Serum sIgEs, skin prick test results and FA diagnosis in infants with moderate-to-severe AD.

Supplementary Table 2. Allergens of ISAC.

Supplementary Table 3. Flow Cytometry panels.

Supplementary Figure 1: Gating strategies for the identification of Treg cells and basophils.

METHODS

Diagnosis criteria and patients´ clinical features

Children with atopic dermatitis (AD) diagnosis who met the requirements to participate in the study were referred by the Primary Care pediatricians to the Pediatrics Service of Cruces University Hospital where the study was carried out. The diagnosis of AD was made according to the diagnostic criteria of the Danish Allergy Research Centre:

- Itching on history or examination
- Chronicity: symptoms in the last 6 weeks
- Location: in 2 of the 4 locations: face, trunk, extensor surfaces of arm and legs.

And the severity criteria were as follows:

- Having received a course of systemic corticosteroids.
- Having used tacrolimus on the advice of a dermatologist.
- Having received more than 7 days of topical corticosteroids in one month.

As mentioned in the main text, food challenge for the FA diagnosis was not justified, given the high predictive value of the degree of sensitization assessed by serum sIgE values and/or prick test results (**Table 1** and **Supplementary Table 1**). The aim of the study was to demonstrate that moderate-to-severe AD is a risk factor for food sensitization and the introduction of new foods. It was considered sufficient to know the sensitization profile and/or prick test results of the children with moderate-to-severe AD and thus avoid food provocation for the benefit of the child.

As the children were breastfed, the foods to which each child was sensitized were eliminated from the mother´s diet. In the only non-sensitized case (DA001), the maternal diet was not modified, and subsequently a diagnosis of non-IgE-mediated egg allergy was made with a clinical picture of severe acute enterocolitis.

Food was only introduced during the follow-up of the children when IgE titres dropped below 2 KU/L and the prick was <3mm.

List of antibodies used for flow cytometric analysis

The following anti-human monoclonal antibodies (mAbs) were used for flow cytometric analysis: FITC anti-CD63 (clone H5C6), BV510 anti-CD123 (clone 9F5) PerCP-Cy5.5 anti-HLA-DR (clone G46-6), BV421 anti-CD192 (CCR2) (clone 48607), BB515 anti-CD127 (clone HIL-7R-M21), PE-Cy7 anti-CD3 (clone SK7), BV421 anti-CD4 (clone RPA-T4), BV421 anti-CD152 (CTLA-4) (clone BNI3) and BV510 anti-CD45RO (clone UCHL1) from BD Biosciences; PE anti-CD300a (clone E59.126) from Beckman-Coulter; eFluor660 anti-CD300c (clone TX45) and PE-Cy7 anti-FcεRI (clone AER-37) from eBioscience; APC-Cy7 anti-CD184 (CXCR4) (clone 12G5), PE anti-CD25 (clone M-A251), PerCP-Cy5.5 anti-CD3 (clone SK7) and APC anti-PD-1 (clone NAT-105) from BioLegend; PerCP-eFluor710 anti-Helios (clone 22F6), PE-Cy7 anti-CD4 (clone RPA-T4), APC anti-Foxp3 (clone PCH101) from Invitrogen.

Supplementary Table 1. Serum sIgEs, skin prick test results and FA diagnosis in infants with moderate-to-severe AD. (*nd: no data available)

Patient	ImmunoCap (>0,35 KU/L) at 2 years	Skin prick test	Diagnosis (2 years old)
DA001	Negative	Ovomucoid (<3mm); ovalbumin (<3mm)	Non IgE-mediated egg allergy
DA003	Ovomucoid (0,03); ovalbumin (1,68); Peanut (0,36); rArah1 (0,41)	Ovomucoid (7mm); ovalbumin (<3mm); peanut (<3mm)	Egg allergy
DA004	Ovomucoid (7,00); cashew nuts (2,36); walnuts (2,62)	Ovomucoid (4mm); cashew nuts (12mm); pistachio (10mm); hazelnut (8mm)	Egg and nuts allergy
DA005	Ovomucoid (5,97); ovalbumin (1,24); peanut (4,45); rAra h2 (4,69); rAra h6 (1,51); hake (12,00); shrimp (1,08); cod (8,81); rGad c 1 (9,62); salmon (3,62); horse mackerel (8,31); roosterfish (7,36); lentils (1,50); chickpeas (1,71)	Ovomucoid (4mm); lentils (4mm); hazelnut (3mm); almond (4mm); pistachio (4mm); pinions (2mm)	Egg, fish, crustaceans, legumes and nuts allergy
DA006	Ovomucoid (44,70); ovalbumin (8,81); cow milk (12,70); α-lactoalbumin (0,38); β-lactoglobulin (0,99); casein (15,00); peanut (16,20); rAra h3 (1,04); rAra h6 (6,36); hazelnut (14,60); almond (10,40); pistachio (8,95); cashew nuts (5,58); wheat (6,27)	nd*	Milk, egg and nuts allergy
DA007	Egg white (12,40); ovomucoid (8,89); ovalbumin (6,66); cow milk (7,47); α-lactoalbumin (1,44); β-lactoglobulin (0,41); casein (1,56); peanut (1,76); rAra h1 (1,04); rAra h9 (1,08); almond (1,75); oat (2,53)	nd*	Milk, egg and nuts allergy
DA008	Cow milk (5,81); α-lactoalbumin (6,23); casein (1,83); ovalbumin (0,55); ovomucoid (1,48); potato (2,62); kiwi (5,03); hake (3,06); cod (1,11); rGad c 1 (2,74); roosterfish (2,82)	Ovomucoid (< 3mm)	Milk, kiwi and fish allergy
DA009	nd*	Ovomucoid (<3mm); ovalbumin (<3mm)	Raw egg allergy (based on clinical symptoms)
DA010	Egg white (19,20); egg yolk (2,68); ovalbumin (9,75); ovomucoid (21,80); cow milk (26); β-lactoglobulin (1,60); casein (38,10) almond (6,39); peanut (0,48); hazelnut (3,93); pistachio (4,80); walnut (0,91); cashew nuts (3,58); wheat (8,51); oat (1,99)	nd*	Milk, egg, wheat and nuts allergy
DA011	nd*	Cow´s milk (<3mm); ovomucoid (<3mm); ovalbumin (<3mm)	Milk and undercooked egg allergy (based on clinical symptoms)

Supplementary Table 2. Allergens of ISAC (*Adapted from Thermo Fisher Scientific*)

			POSSIBLE CROSS REACTIVITY																				
SOURCE	ALLERGEN	Mainly species-specific proteins de especie	Fruits	Vegetables	Nuts,	Legumes	Cereals	Spices	Grass	Tree pollen	Weed	Latex	Milk	Meat	Fish	Egg	Seafood	Animals	Fungi	Mites	Insects	Venoms	Parasites
Egg white	nGal d 1															•							
Egg white	nGal d 2															•							
Egg white	nGal d 3															•							
Egg yolk/chicken	nGal d 5													•		•							
Cow´s milk	nBos d 4												•										
Cow´s milk	nBos d 5												•										
Cow´s milk and meat	nBos d 6												•	•				•					
Cow´s milk	nBos d 8												•										
Cow´s milk	nBos d Lactoferrina												•										
Cod	rGad c 1														•								
Shrimp	nPen m 1																•			•	•		•
Shrimp	nPen m 2																•				•		
Shrimp	nPen m 4																•						
Cashew nut	rAna o 2	•																					
Brazil nut	rBer e 1	•																					
Hazelnut	rCor a 1.0401		•	•	•	•				•													
Hazelnut	rCor a 8		•	•	•	•	•	•		•	•												
Hazelnut	nCor a 9	•																					
Walnut	rJug r 1	•																					
Walnut	nJug r 2	•																					
Walnut	nJug r 3		•	•	•	•	•	•		•	•												
Sesame	nSes i 1	•																					
Peanut	rAra h 1	•																					
Peanut	rAra h 2	•																					
Peanut	rAra h 3	•																					
Peanut	nAra h 6	•																					
Peanut	rAra h 8		•	•	•	•				•													
Peanut	rAra h 9		•	•	•	•	•	•		•	•												
Soy	rGly m 4		•	•	•	•				•													
Soy	nGly m 5	•																					
Soy	nGly m 6	•																					
Buckwheat	nFag e 2	•																					
Wheat	rTri a 14		•	•	•	•	•	•															
Wheat	rTri a 19	•																					
Wheat	nTri a aA_TI						•																
Kiwi	nAct d 1	•																					
Kiwi	nAct d 2		•																•	•			
Kiwi	nAct d 5	•																					
Kiwi	rAct d 8		•	•	•	•				•													
Celery	rApi g 1		•	•	•	•				•													
Apple	rMal d 1		•	•	•	•				•													
Peach	rPru p 1		•	•	•	•				•													
Peach	rPru p 3		•	•	•	•	•	•		•	•												
Timothyme grass	rPhl p 1								•														
Timothyme grass	rPhl p 2								•														
Timothyme grass	nPhl p 4								•														
Timothyme grass	rPhl p 5								•														
Timothyme grass	rPhl p 6								•														
Timothyme grass	rPhl p 7								•	•	•												
Timothyme grass	rPhl p 11								•														
Timothyme grass	rPhl p 12		•	•	•	•	•	•	•	•	•	•											
Alder	rAln g 1		•	•	•	•				•													
Birch	rBet v 1		•	•	•	•				•													
Birch	rBet v 2		•	•	•	•	•	•	•	•	•	•											
Birch	rBet v 4								•	•	•												
Hazel	rCor a 1,0101		•	•	•	•				•													
Japanese cedar	nCry j 1	•																					
Cypress	nCup a 1	•																					
Common couch grass	nCyn d 1																						
Olive tree	rOle e 1	•							•														
Olive tree	nOle e 7		•	•	•	•	•	•		•	•												
Olive tree	rOle e 9	•																					
Shade banana	rPla a 1	•																					
Shade banana	nPla a 2	•																					
Shade banana	rPla a 3		•	•	•	•	•	•		•	•												
Ragweed	nAmb a 1	•																					
Artemisia	nArt v 1	•																					
Artemisia	nArt v 3		•	•	•	•	•	•		•	•												
Ashy	rChe a 1	•																					
Mercurial	rMer a 1		•	•	•	•	•	•	•	•	•	•											
Parietaria	rPar j 2	•																					
Plantain	rPla l 1	•																					
Salsola	nSal k 1	•																					
Dog	rCan f 1																	■					
Dog	rCan f 2	•																					
Dog	nCan f 3												•	•				•					
Dog	rCan f 5	•																					
Horse	rEqu c 1																	■					
Horse	nEqu c 3												•	•				•					
Cat	rFel d 1	•																					
Cat	nFel d 2												•	•				•					
Cat	rFel d 4																	■					
Mouse	nMus m 1																	■					
Alternaria	rAlt a 1	•																					
Alternaria	rAlt a 6																		•				
Aspergillus fumigatus	rAsp f 1	•																					
Aspergillus fumigatus	rAsp f 3																		•				
Aspergillus fumigatus	rAsp f 6																		•				
Aspergillus fumigatus	rAsp f 8																		•				
Blomia	rBlo t 5	•																					
Dermatophagoides	nDer f 1																			•			
Dermatophagoides	rDer f 2																			•			
Dermatophagoides	nDer p 1																			•			
Dermatophagoides	rDer p 2																			•			
Dermatophagoides	rDer p 10																•			•	•		•
Lepidoglyphus	rLep d 2	•																					
Cockroach	rBla g 1	•																					
Cockroach	rBla g 2	•																					
Cockroach	rBla g 5	•																					
Cockroach	nBla g 7																•			•	•		•
Bee	rApi m 1	•																					
Bee	nApi m 4	•																					
Polistes	rPol d 5	•																					
Vespula	rVes v 5	•																					
Anisakis	rAni s 1	•																					
Anisakis	rAni s 3																•			•	•		•
Latex	rHev b 1	•																					
Latex	rHev b 3	•																					
Latex	rHev b 5	•																					
Latex	rHev b 6.01		•									•											
Latex	rHev b 8		•	•	•	•	•	•	•	•	•	•											
Bromelain	nMUXF3		•	•	•	•	•	•	•	•	•	•					•				•	•	•

Supplementary Table 3. Flow Cytometry panels

	FITC	PE	PerCP-Cy5.5	PE-Cy7	APC	APC-Cy7	BV421	BV510
**Basophils**	CD63	CD300a	HLA-DR	FcεRI	CD300c	CXCR4	CCR2	CD123
**Treg cells_1**	CD127	CD25	Helios	CD3	Foxp3	Viability	CD4	CD45RO
**Treg cells_2**	CD127	CD25	CD3	CD4	PD-1	Viability	CTLA-4	

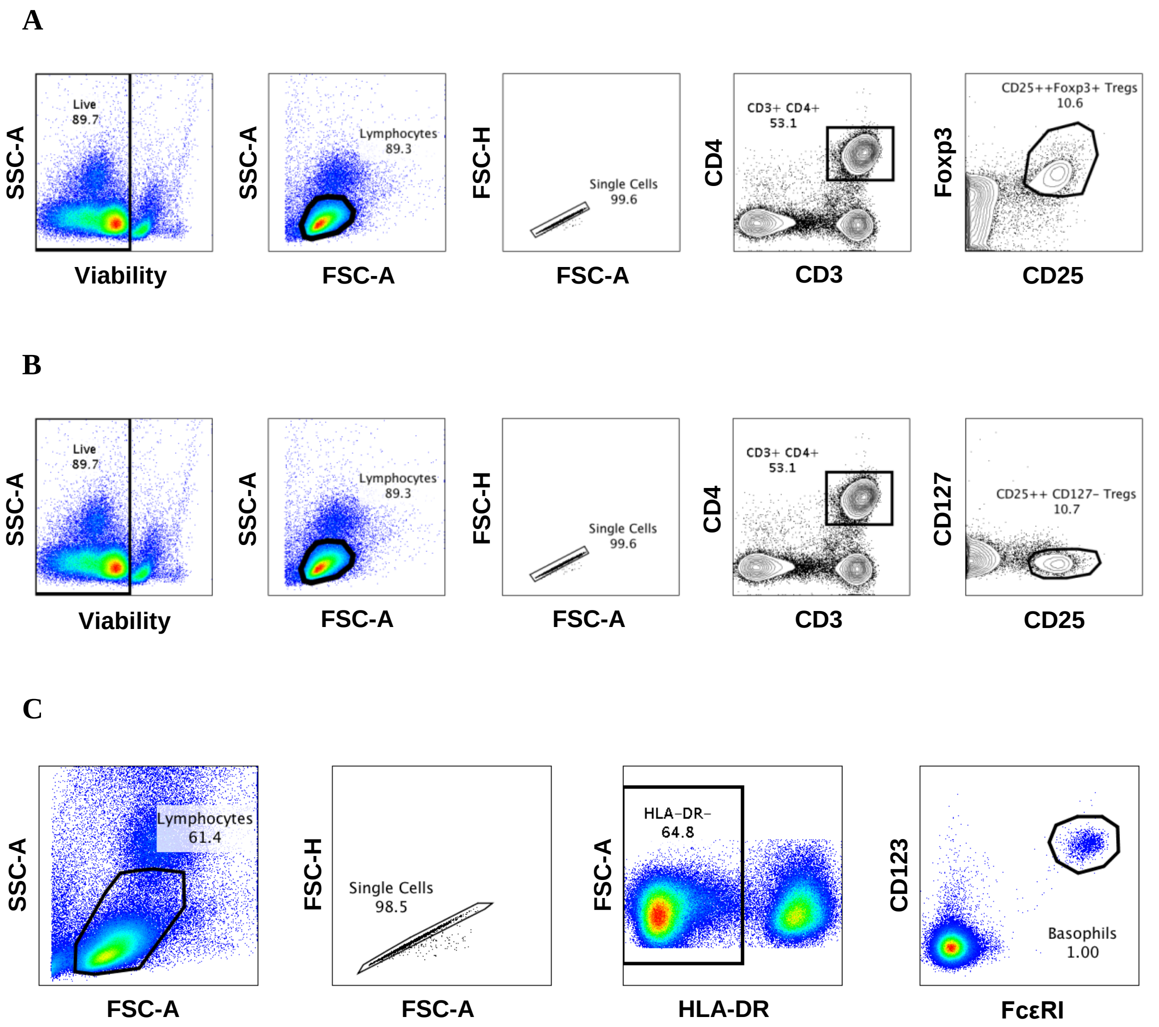

Supplementary Figure 1: Gating strategies for the identification of Treg cells and basophils. Pseudocolor and contour plots representing the gating strategy utilized for the identification of Treg cells **(A, B)** and basophils **(C)**. **(A, B)** Two gating strategies were used to identify Treg cells among viable PBMCs. On the one hand, they were identified as CD25^{high}FoxP3+ cells within CD3+CD4+ single cells **(A)**. On the other hand, the identification was made based on the high expression of CD25 and the absence of CD127 within CD3+CD4+ single cells **(B)**. **(C)** Basophils were identified based on the expression of FcεRI and CD123 within HLA-DR negative single cells.
